# Analysis of the Ribonuclease A Superfamily of Antimicrobial Peptides in Patients Undergoing Chronic Peritoneal Dialysis

**DOI:** 10.1038/s41598-019-44219-x

**Published:** 2019-05-23

**Authors:** Neha Dhingra Pottanat, Amy C. Brook, Maria Bartosova, Hanna Cortado, Sudipti Gupta, Birong Li, Ashley R. Jackson, Martin Vonau, Shira Cohen, Maria Ferrara, Christina B. Ching, John David Spencer, Annelie Brauner, Donald J. Fraser, Claus Peter Schmitt, Matthias Eberl, Rose Ayoob, Brian Becknell

**Affiliations:** 1 Division of Nephrology, Department of Pediatrics, Riley Children’s Hospital and Indiana University School of Medicine, Indianapolis, Indiana USA; 2 Division of Infection and Immunity, School of Medicine and Systems Immunity Research Institute, Cardiff University, Cardiff, CF14 4XN United Kingdom; 3 Division of Pediatric Nephrology, Center for Pediatric and Adolescent Medicine, University of Heidelberg, Heidelberg, Germany; 4 Center for Clinical and Translational Research, The Research Institute at Nationwide Children’s Hospital, Columbus, OH USA; 5 Department of Pediatrics and Internal Medicine, Ohio State University College of Medicine, Columbus, OH USA; 6 Division of Neonatology, Department of Pediatrics, Nationwide Children’s Hospital, Columbus, OH USA; 7 Division of Pediatric Urology, Department of Surgery, Nationwide Children’s Hospital, Columbus, OH USA; 8 Division of Nephrology, Department of Pediatrics, Nationwide Children’s Hospital, Columbus, OH USA; 9 Department of Microbiology, Tumor and Cell Biology, Division of Clinical Microbiology, Karolinska Institutet and Karolinska University Hospital, Stockholm, Sweden; 10 Wales Kidney Research Unit, Cardiff University, Cardiff, United Kingdom; 11 Division of Nephrology, Department of Pediatrics, Charleston, WV USA

**Keywords:** Peritoneal dialysis, Antimicrobial responses

## Abstract

Infectious peritonitis is a common complication in patients undergoing chronic peritoneal dialysis (PD), limiting the duration of PD as a modality for renal replacement therapy and increasing patient morbidity and mortality. Antimicrobial peptides (AMPs) serve critical roles in mucosal defense, but their expression and activity during peritonitis are poorly understood. We hypothesized that AMPs belonging to the Ribonuclease (RNase) A Superfamily are present in peritoneal fluid and increase during peritonitis in patients undergoing chronic PD. In the absence of peritonitis, we detected RNase 3, RNase 6, and RNase 7 in cell-free supernatants and viable cells obtained from peritoneal fluid of chronic PD patients. The cellular sources of these RNases were eosinophils (RNase 3), macrophages (RNase 6), and mesothelial cells (RNase 7). During peritonitis, RNase 3 increased 55-fold and RNase 7 levels increased 3-fold on average, whereas RNase 6 levels were unchanged. The areas under the receiver-operating characteristic curves for RNase 3 and RNase 7 were 0.99 (95% confidence interval (CI): 0.96–1.0) and 0.79 (95% CI: 0.64–0.93), respectively, indicating their potential as biomarkers of peritonitis. Discrete omental reservoirs of these RNases were evident in patients with end stage kidney disease prior to PD initiation, and omental RNase 3 reactive cells increased in patients undergoing PD with a history of peritonitis. We propose that constitutive and inducible pools of antimicrobial RNases form a network to shield the peritoneal cavity from microbial invasion in patients undergoing chronic PD.

## Introduction

Peritoneal dialysis (PD) is a well-established modality of chronic renal replacement therapy worldwide, accounting for 11% of the overall dialysis population^1^. The duration of chronic PD is frequently limited by episodes of infectious peritonitis. Several risk factors leading to peritonitis have been addressed by modifying PD catheter design and surgical techniques during catheter insertion^2^. Additional changes in connection techniques, catheter exit-site care, and patient training have reduced peritonitis rates over the last decade^3–5^. Despite these improvements, peritonitis is a principal reason for hospitalization, conversion to hemodialysis, and death in PD patients^6,7^. In a recent study, preventing PD-related infections was identified as the top priority for PD patients and their caregivers^8^.

Further reductions in peritonitis rates may be achieved through strategies that enhance peritoneal innate immune defenses against invading microorganisms. Devising such strategies requires a more complete understanding of peritoneal defense mechanisms and how they are influenced by chronic kidney disease (CKD), PD, and peritonitis. The peritoneum is lined by mesothelial cells that possess innate defense mechanisms including: barrier function, constitutive secretion of antimicrobial mediators, and expression of pattern recognition receptors that serve as microbial sensors^9–11^. Mesothelial cells are assisted by patrolling leukocytes, such as eosinophils and macrophages, and together these cells form an interactive network to coordinate a rapid and efficient innate immune response^12,13^.

Innate immune cells utilize a variety of antibacterial peptides and proteins to neutralize microbial invaders, including antimicrobial peptides (AMPs)^14,15^. AMPs may serve as novel antimicrobial agents^16,17^. In addition to their antimicrobial action, AMPs modulate the activity of epithelial and inflammatory cells, influencing diverse processes such as mitosis, wound healing, cytokine release, chemotaxis, protease-antiprotease balance, and redox homeostasis^18^. Defensins, Cathelicidin (LL-37), and Neutrophil Gelatinase-Associated Lipocalin (NGAL) are examples of AMPs that are present in the peritoneal cavity^19–22^.

The Ribonuclease (RNase) A Superfamily encodes multiple AMPs with potent microbicidal activity against Gram-positive, Gram-negative, and fungal organisms^23–27^. These antimicrobial RNases exhibit cell-specific expression patterns. Whereas RNase 3 and RNase 6 are produced by leukocytes, RNase 7 is constitutively secreted by epithelial cells of the skin, airway, and urinary tract^23,28^. In this study, we hypothesized that these antimicrobial RNases are present in the peritoneal cavity of patients undergoing chronic PD, where they are produced by specific cell populations and exhibit unique changes in expression following peritonitis.

## Results

### RNase 3,6, and 7 are present in peritoneal fluid of patients on chronic PD and differentially regulated during peritonitis

**Figure 1:**
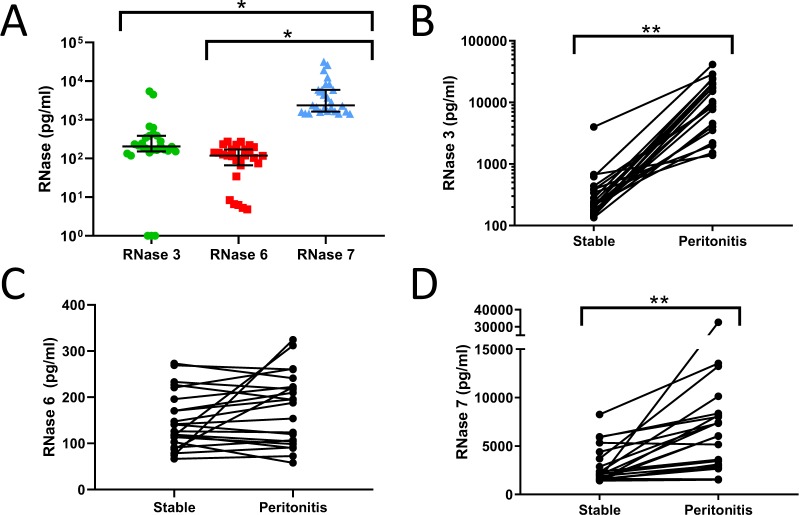
Peritoneal fluid RNase concentrations in chronic PD patients in the absence of infection and following peritonitis. (**A**) In 27 uninfected, chronic PD patients, RNase 7 concentrations were increased, compared to RNase 3 and RNase 6. Lines indicate median and interquartile range. *Adjusted *p* < 0.0001, Kruskal-Wallis test with Dunn’s correction for multiple comparisons. (**B**–**D**) RNase 3, RNase 6, and RNase 7 concentrations in 22 PD patients with peritonitis. Each patient’s RNase concentration during peritonitis is compared to that obtained in the absence of infection (**p < 0.0001, Wilcoxon matched-pairs signed rank test).

**Figure 2:**
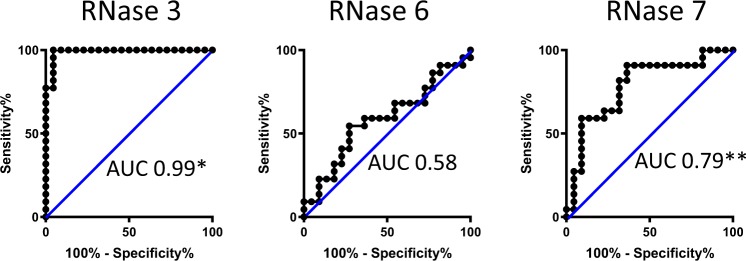
ROC curves identify RNase 3 and RNase 7 as potential biomarkers of peritonitis. AUC: Area under the ROC curve. *p < 0.0001 and **p = 0.0012.

We investigated peritoneal fluid concentrations of RNase 3, 6, and 7 in a cohort of 27 patients undergoing chronic PD, comprising 21 adult and six pediatric patients (Table S1). At the time of peritoneal fluid collection, all patients lacked signs and symptoms of peritonitis. No patient experienced a peritonitis episode within the month preceding or following peritoneal fluid collection. RNase concentrations were measured in peritoneal fluid by enzyme linked immunosorbent assay (ELISA). All three antimicrobial RNases were detectable in all patients, with the exception of three pediatric patients who lacked RNase 3 protein expression (Fig. 1A). RNase 7 levels were significantly higher than RNase 3 or RNase 6 (Fig. 1A). Compared to adult patients, pediatric RNase 6 levels were lower and RNase 7 levels were higher (Fig. S1). Next, RNase concentrations were measured in peritoneal fluid samples from 21 adults and one pediatric patient with peritonitis. Concentrations were compared to uninfected samples collected from the same individuals. During peritonitis, RNase 3 levels increased 55-fold on average (range 2–134), from a median value of 211 pg/ml (IQR 167–385) in uninfected fluid to 9977 pg/ml (IQR 3906–20,332) in peritonitis (Fig. 1B). In five adult patients who experienced recurrent peritonitis, RNase 3 concentration returned to uninfected levels when measured >3 months post-infection and subsequently increased with the next peritonitis episode (Fig. S2). RNase 6 levels did not vary significantly between uninfected and peritonitis samples (Fig. 1C). RNase 7 levels increased 3-fold on average (range 1–19), from a median value of 2071 pg/ml (IQR 1592–4628) in uninfected fluid to 6692 pg/ml (IQR 3080–8805) in adults with peritonitis (Fig. 1D). The areas under the receiver operator characteristics (ROC) curves for RNase 3 and RNase 7 were 0.99 (95% CI, 0.97–1.0) and 0.79 (95% CI, 0.64–0.93), respectively (Fig. 2). There was no statistically significant relationship between RNase levels and the outcome of peritoneal fluid culture (i.e., culture negative, Gram-negative, or Gram-positive bacteria) in patients with peritonitis (data not shown).

### Cellular sources of RNases in peritoneal fluid

**Figure 3:**
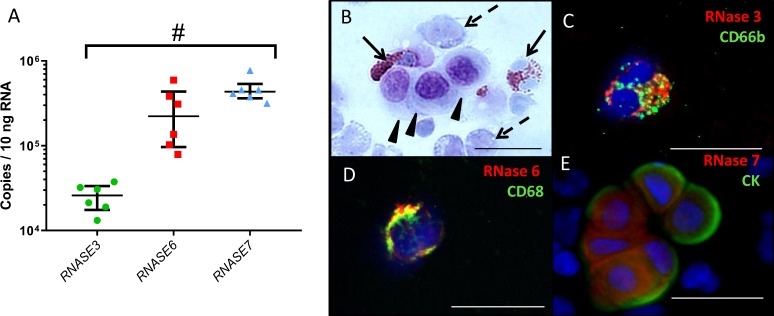
RNase expression by cells obtained from peritoneal fluid of uninfected pediatric PD patients. (**A**) *RNASE3*, *RNASE6*, and *RNASE7* mRNA levels. Lines indicate median and interquartile range. ^#^*p* = 0.0013; Kruskal-Wallis test with Dunn’s correction for multiple comparisons. (**B**–**E**) RNases exhibit cell-specific distribution in peritoneal fluid. The initial drain was collected prior to starting nightly CCPD and subject to cytocentrifugation. (**B**) Kwik-Diff staining identifies eosinophils (solid arrow), macrophages (dashed arrows) and mesothelial cells (arrowheads) in peritoneal fluid; (**C**) Granular distribution of RNase 3 in a CD66b(+) eosinophil; (**D**) RNase 6 reactivity and partial co-localization with CD68, a macrophage lineage marker; (**E**) Cytoplasmic staining of RNase 7 in a Cytokeratin (CK)(+) cluster of mesothelial cells. All figures are 40x original magnification. Scale bar: 25 microns. Representative images from at least 3 separate patients are shown.

We hypothesized that the presence of RNases in uninfected peritoneal fluid may be due to their local production by cells within the peritoneal cavity. We therefore isolated viable cells from peritoneal fluid of six pediatric PD patients and measured *RNASE3*, *RNASE6*, and *RNASE7* mRNA levels by quantitative reverse transcriptase polymerase chain reaction (qRT-PCR). Pediatric patients are particularly well suited for these studies for two reasons: (1) Their underlying diseases are mostly restricted to the kidney and urinary tract; and (2) Children lack secondary pathologies often present in adults, such as changes related to long-standing hypertension, diabetes, smoking, and aging. In all cases, *RNASE* mRNA expression was detectable, and *RNASE7* mRNA levels were more abundant than *RNASE3* (Fig. 3A). We evaluated RNase protein expression by immunofluorescence microscopy and performed co-staining with lineage specific antibodies. Using a differential staining method, we detected mesothelial cells, monocytes, and eosinophils in peritoneal fluid from these patients (Fig. 3B). We found that RNase 3, RNase 6, and RNase 7 localized exclusively to CD66b(+) eosinophils, CD68(+) monocytes, and Cytokeratin (CK)(+) mesothelial cells, respectively (Fig. 3C–E).

### RNases localize to discrete cell types within human omentum

**Figure 4:**
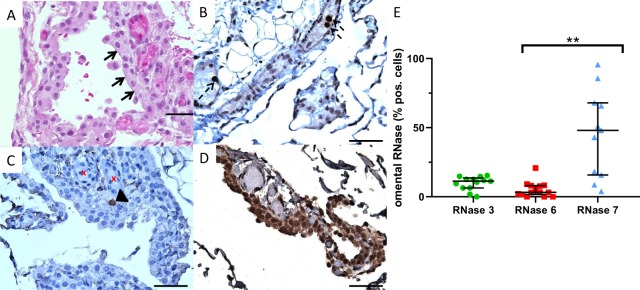
Localization of RNases in omentum from children with stage 5 CKD. (**A**) H&E staining illustrates normal omental morphology. Arrows indicate mesothelium. Abundant capillaries are evident within the mesothelium. (**B**) RNase 3 is expressed by leukocytes (dashed arrows) in omental blood vessels. (**C**) RNase 6 is expressed by rare interstitial cells (black arrowhead) in the submesothelial space near blood vessels (x). (**D**) RNase 7 is expressed by mesothelial cells. Scales bars indicate 32.5 microns (**A**) and 50 microns (**B**–**D**). Original magnification 60x (**A**) and 40x (**B**–**D**). Representative micrographs from 4 patients are shown. (**E**) Quantification of omental RNase+ cells. **p < 0.0001 Kruskal-Wallis test with Dunn’s correction for multiple comparisons.

We investigated RNase distribution by immunohistochemistry in omentum from children with stage 5 CKD at the time of PD catheter insertion. The omentum is a highly vascularized structure composed of fibroadipose tissue that is lined with mesothelial cells (Fig. 4A). RNase 3 localized to leukocytes within omental blood vessels (Fig. 4B), while RNase 6 reactivity was restricted to occasional cells in the submesothelial space (Fig. 4C). RNase 7 uniquely localized to the mesothelial lining (Fig. 4D).

**Figure 5:**
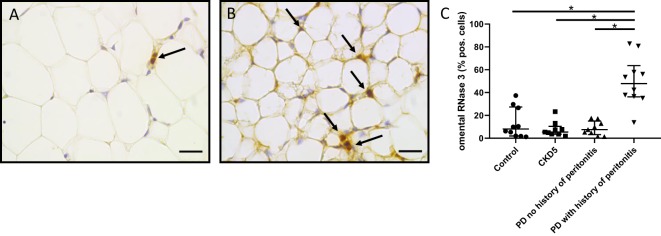
Impact of peritonitis on RNase 3 expression in pediatric omentum. (**A**) Sparse omental RNase 3+ cells (arrow) in PD patients without a history of peritonitis. (**B**) Widespread omental RNase 3+ cells following peritonitis (arrows). Both micrographs are 20x magnification. Scale bars indicate 20 μm. (**C**) Quantification of RNase 3+ cell frequency in children undergoing PD with a history of peritonitis, compared to indicated control populations (*p < 0.0001, Kruskal-Wallis test with Dunn’s correction for multiple comparisons; 9–10 patients/group).

Since RNase 3 exhibited the greatest induction in peritoneal fluid during peritonitis (Fig. 1B), we also evaluated the impact of peritonitis on the proportion of RNase 3 immunoreactive cells in omentum. For these studies, we utilized a registry of omental tissue from pediatric patients with normal kidney function, stage 5 CKD prior to dialysis initiation, and chronic PD with and without a history of peritonitis^29^. Using automated quantitative immunohistochemistry, we determined that omentum from patients with a history of peritonitis contained a greater proportion of RNase 3+ cells, compared to the other experimental groups (Fig. 5).

## Discussion

AMPs in the RNase A Superfamily exhibit potent, broad-spectrum antimicrobial activity toward microorganisms implicated in peritonitis in the chronic PD population^5,25,26,30^. In this study, we demonstrated the presence of RNase 3, RNase 6, and RNase 7 in the peritoneal fluid of patients receiving chronic PD who lacked clinical signs and symptoms of peritonitis. During peritonitis, we observed accumulation of RNase 3 and RNase 7 in peritoneal fluid. Immunolocalization studies in peritoneal fluid and omentum revealed distinct cellular reservoirs for each RNase. Collectively, these findings suggest roles for antimicrobial RNases in promoting antimicrobial immunity in patients undergoing chronic PD.

### RNase 3 levels increase during peritonitis

Among the antimicrobial RNases evaluated in this study, RNase 3 exhibited the most consistent induction during peritonitis. RNase 3 induction occurred regardless of culture results, suggesting that the release of RNase 3 may be elicited by the host immune response rather than in response to a specific microbial cue. This is supported by a study that demonstrated RNase 3 release by eosinophils in response to cytokine priming and the addition of platelet activating factor or complement factor C5a^31^. Increased RNase 3 levels may also reflect the degranulation or recruitment of eosinophils in PD patients with peritonitis, which contain large amounts of RNase 3 in cytoplasmic granules^32^. Neutrophils represent an alternative reservoir of RNase 3, and their brisk recruitment and degranulation may account for increased RNase 3 levels in peritonitis^33–35^. The average concentration of RNase 3 in infected peritoneal fluid is 3–4 orders of magnitude below the minimal inhibitory concentration of recombinant RNase 3 toward Gram-positive and Gram-negative bacteria (H.C. and B.B., unpublished observations)^36,37^. It is therefore unlikely that RNase 3 exerts significant bactericidal activity in peritoneal fluid. Rather, we and others have hypothesized that RNase 3 exerts antimicrobial activity intracellularly, i.e., toward ingested bacteria within the eosinophil’s phagosome^28^. Alternatively, it is possible that RNase 3 serves an immunomodulatory function. RNase 3 exhibits high-affinity binding to bacterial cell wall components such as peptidoglycan and lipopolysaccharide^38^. This binding neutralizes bacterial endotoxin and limits pro-inflammatory cytokine production by phagocytes^39^. This raises the possibility that increased RNase 3 levels may serve to dampen the local immune response and prevent septic shock syndrome. Further studies are required to clarify the biological significance of RNase 3 during peritonitis.

### Mesothelial cells are a novel source of RNase 7

Whereas leukocyte populations appear to account for limited RNase 3 and RNase 6 expression in peritoneal fluid, mesothelial cells serve as the sole source of RNase 7, which is the most abundant of the antimicrobial RNases evaluated in this study. The production of RNase 7 by mesothelial cells is consistent with studies in the skin, airway, and urinary tract, which have demonstrated constitutive secretion of RNase7 by epithelial cells^24,40–42^. These studies have led to the hypothesis that RNase 7 serves as an antimicrobial shield to prevent epithelial attachment and invasion by microorganisms^43^. Mesothelial cells express pattern recognition receptors and rapidly synthesize cytokines, chemokines, and AMPs in response to microbial exposure^44,45^. Chronic PD leads to mesothelial cell loss, and this depletion of mesothelial cells may lead to intraperitoneal deficiency in RNase 7 and increased peritonitis susceptibility^46,47^. Mesothelial loss as a consequence of chronic PD may account for absent induction of RNase 7 in certain patients with peritonitis. It is also noteworthy that RNase 7 levels were higher in children than adults in this study. The potential links between RNase 7 levels, patient age, mesothelial content, and peritonitis susceptibility warrant additional investigation.

### Diagnostic utility of RNase 3 and RNase 7 as peritonitis biomarkers

The areas under the ROC curves for RNase 7 and particularly RNase 3 predict that they are good to excellent candidate biomarkers of peritonitis. Peritoneal fluid cell count, differential, gram stain, and culture are the current standard evaluation tools to diagnosis peritonitis in patients receiving chronic PD. Each dialysis center is required to maintain a culture-negative peritonitis rate of <20% to insure that the culture technique is adequate^7^. This highlights the attention to detail that dialysis centers need to maintain when evaluating patients with peritonitis and the need for improved diagnostic testing. Further, larger scale studies are required to determine whether the addition of RNase 3 and RNase 7 measurement to standard evaluation methods increases the sensitivity and specificity of peritonitis diagnosis in the chronic PD patient population.

### Omentum is a rich source of RNase expressing cells

In this study, we implicate omental mesothelium and leukocytes as distinct cellular sources of AMPs belonging to the RNase A Superfamily. The RNases join a growing number of AMPs expressed by omentum, as omental adipocytes serve as sources of AMPs such as defensins and cathelicidin^48,49^. When omentum was examined in patients with a history of peritonitis, we observed a greater proportion of RNase 3+ cells – even in instances where tissue was harvested 24 weeks after peritonitis. In this way, the omentum serves as a local reservoir of RNase 3+ cells that can rapidly mobilize in response to subsequent microbial exposure. The omentum is well-suited for this purpose, as omental high endothelial venules promote rapid neutrophil recruitment that protects mice with peritonitis from developing sepsis, and omental milky spots concentrate invading microorganisms where they are engulfed and killed by phagocytes^50^.

These antimicrobial properties of omentum are especially intriguing in the pediatric chronic PD population, as the six pediatric patients in our study had either a partial or complete omentectomy at the time of PD catheter placement. Omentectomy is commonly performed in pediatric patients to reduce the risk of PD catheter malfunction^51^. Furthermore, pediatric PD patients represent a unique patient group to investigate the impact of PD and PD-associated peritonitis on the omentum, as the vast majority suffer from non-inflammatory diseases, mainly congenital urinary tract malformations. This lack of baseline inflammation increases the likelihood of a causal link between the observed increase in omental RNase 3+ cells and history of peritonitis in these patients.

## Conclusions

**Figure 6:**
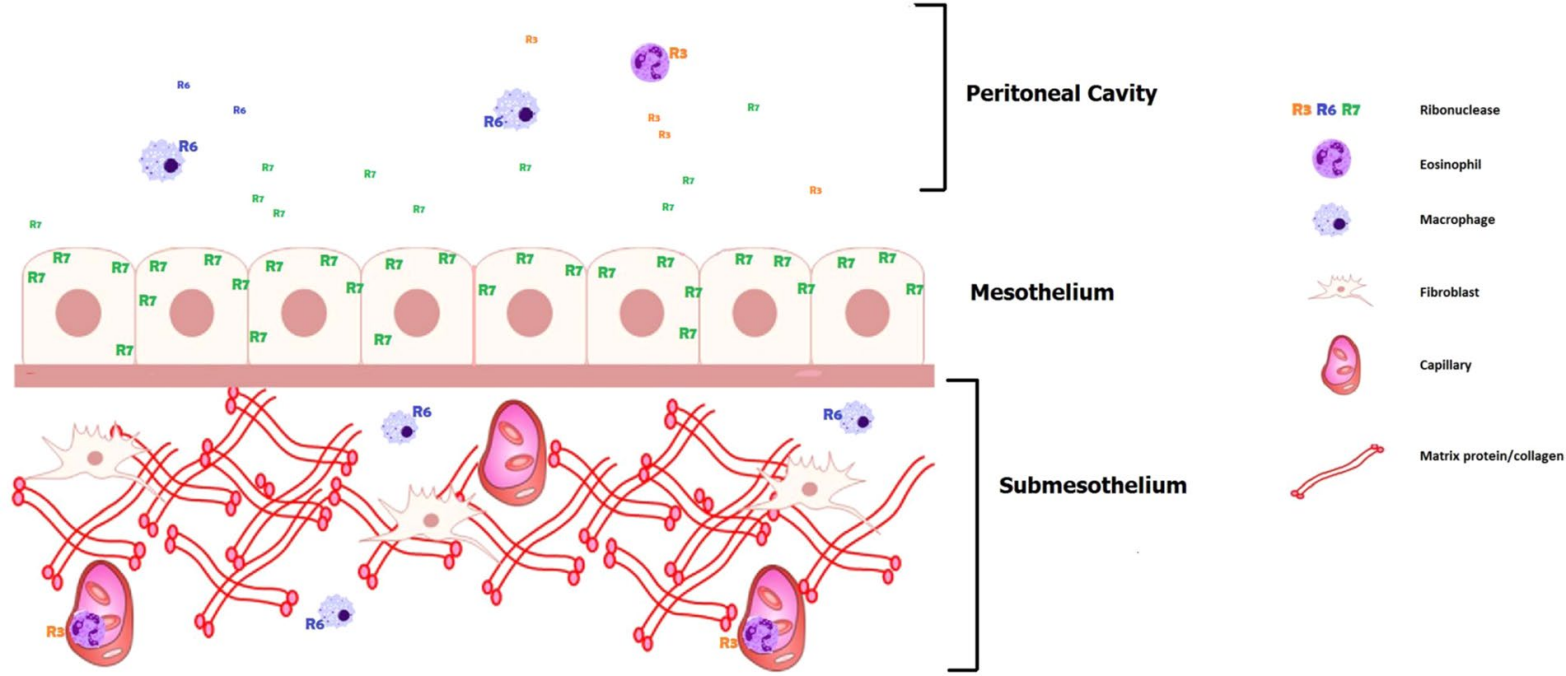
A network of RNases protects the peritoneum from microbial invasion. Mesothelial cells constitutively secrete RNase 7 (R7). Macrophages patrol the submesothelial space and synthesize RNase 6 (R6). Eosinophils circulate within the peritoneal microvasculature and produce RNase 3 (R3). In addition, both R3+ eosinophils and R6+ macrophages patrol the peritoneal cavity.

We have demonstrated the presence of multiple antimicrobial RNases with distinct cellular sources in the peritoneal fluid of patients undergoing chronic PD, which display unique expression patterns following peritonitis. Peritoneal fluid concentrations of RNase 3 and RNase 7 increase during peritonitis, and further studies are warranted to test their efficacy as peritonitis biomarkers. Our data support a model in which a network of RNases helps to protect the peritoneal cavity from microbial invaders (Fig. 6). Mesothelial cells constitutively synthesize RNase 7, which acts as an antimicrobial shield at the surface of the peritoneal membrane. Sentinel leukocytes patrolling the peritoneal cavity and omentum express RNase 3 (eosinophils) and RNase 6 (monocytes/macrophages). We predict that this local antimicrobial RNase network contributes substantially to the prevention and eradication of infectious peritonitis in the PD population.

## Methods

### Adult PD patients

This study was approved by the South East Wales Local Ethics Committee (04WSE04/27) and registered on the UK Clinical Research Network Study Portfolio under reference number #11838 “Patient immune responses to infection in PD”. All individuals provided written informed consent. All research was performed in accordance with relevant guidelines and regulations. 21 adults receiving continuous ambulatory PD (CAPD) or continuous cycling PD (CCPD) at the University Hospital of Wales, Cardiff, between November 2013 and March 2018 were followed for up to three years (Samples 1–21 in Table S1). Cell-free peritoneal effluent samples from ≥8 hour dwells were collected when the individuals were stable (with no infection in the previous month), and within 24 hours of presentation with acute peritonitis, before starting antibiotic treatment. Clinical diagnosis of acute peritonitis was based on the International Society of Peritoneal Dialysis (ISPD) Consensus Guidelines^52^. Patients experienced 1–5 episodes of peritonitis during the study period. Three peritonitis episodes were defined as culture-negative (after incubation of up to 5 days), and the remaining episodes were confirmed bacterial infections. Causative microorganisms are shown in Table S1. Cases of fungal infection and polymicrobial or unclear culture results were excluded from the study.

### Pediatric PD patients

Following approval of the Nationwide Children’s Hospital Institutional Review Board (Protocol IRB16-00086) and provision of informed consent from a parent and/or legal guardian, we recruited six chronic PD patients at Nationwide Children’s Hospital between March 2016 and September 2017 (samples 22–27 in Table S1). All research was performed in accordance with relevant guidelines and regulations. All patients underwent CCPD using dextrose-based (high-GDP) PD solution (Dianeal, Baxter, Deerfield, IL). Peritoneal fluid was collected from the patients after a long (≥8 hours) daytime dwell cycle prior to CCPD. All patients lacked signs and symptoms of peritonitis within the month preceding sample collection. Clinical diagnosis of acute peritonitis was based on the International Society of Peritoneal Dialysis (ISPD) Consensus Guidelines^52^.

### Peritoneal fluid processing

Following centrifugation of peritoneal fluid (500 *g*, 10 minutes, 4 °C), the supernatant was stored at −80 °C for ELISA. The cellular pellet was resuspended in sterile phosphate buffered saline, and enumerated. Over 95% of cells recovered from PD fluid were viable based on trypan blue exclusion. The total number of live cells varied between 50,000 to 200,000. Cells were subsequently lysed in RNA or protein extraction buffer and frozen at −80 °C.

### Pediatric omentum

Formalin-fixed, paraffin-embedded, deidentified human omentum from children with stage 5 CKD was provided with approval from the Nationwide Children’s Hospital Institutional Review Board in collaboration with the Department of Anatomic Pathology (Dr. Peter Baker). Alternatively, as designated in the text, omental samples from children with normal renal function (control), CKD5 at the time of PD start and PD were obtained from the International Pediatric Biopsy Registry (www.clinicaltrials.gov NCT01893710). The groups were age-matched. All patients received neutral pH, low GDP PD fluids. Peritonitis was caused by *Staphylococcus aureus* (6 cases), *Enterobacter* species (1 case), *Corynebacterium* species (1 case), and 2 cases were culture-negative.

### qRT-PCR

Total RNA was extracted from using the RNeasy Mini Kit (Qiagen). RNA was quantified based on absorbance at 260 nm and reverse transcribed into complementary (c)DNA with the Verso cDNA Synthesis Kit (Thermo Fisher Scientific, Waltham, MA) in 20 µl total volume. The cDNA was diluted with 40 µl sterile water, and 2 µl was used as template in qPCR reactions using SybrGreen detection and the following gene-specific primers: *RNASE3* Forward 5′-AGA GAC TGG GAA ACA TGG-3′; *RNASE3* Reverse 5′-GAT AAT TGT TAA TTG CCC GC-3′; *RNASE6* Forward 5′-AGC CCC AAC ACT GAG ACC AGA AAA-3′; *RNASE6* Reverse 5′-GGT GGC AGT TGT GCC GAC GA-3′; *RNASE7* Forward 5′-AAG ACC AAG CGC AAA GCG AC-3′, and *RNASE7* Reverse 5′-GCA GGC TAT TTT GGG GGT CT-3′. Exclusion of reverse transcriptase during cDNA synthesis resulted in undetectable amplification, attesting to amplicon derivation from cDNA rather than contaminating genomic DNA. Positive controls consisted of bone marrow cDNA (*RNASE3*, *RNASE6*) and skin cDNA (*RNASE7*). Amplicons were cloned into pCR4 (Invitrogen, Carlsbad, CA) and bidirectionally sequenced. Standard curves for each amplicon were included with each set of reactions. Absolute transcript levels were expressed per 10 ng total input RNA^23^.

### ELISA

Commercial ELISAs were used for detection of RNase 3 (MBL International, Woburn, MA), RNase 6 (Cloud-Clone), and RNase 7 (Cloud-Clone) in cell-free supernatants of peritoneal fluid. The supplied protocol was followed without modification. Samples were run in triplicate. Only values falling on the standard curve were used. The lower limit of detection was 125 pg/ml (RNase 3), 6.5 pg/ml (RNase 6), and 540 pg/ml (RNase 7).

### Cytology and Immunocytochemistry

50,000 cells were subject to cytocentrifugation. Slides were differentially stained (Kwik-Diff, ThermoFisher Scientific, Waltham, MA). For immunostaining, cells were permeabilized with 100% cold acetone for 2 minutes at room temperature. Slides were washed in phosphate buffered saline (PBS) with 0.05% Tween-20, treated 10 minutes with Superblock (Scytek, Logan, UT), and incubated with primary antibodies overnight at room temperature: α-RNase3 (Abcam), α-RNase6 (Cloud-Clone), α-RNase7 (Sigma), α-CD66b (Stem Cell Technologies, Vancouver, BC), α-CD68 (Stem Cell Technologies), and α-Cytokeratin (CAM5.2, Becton Dickinson, Franklin Lakes, NJ). The next day, slides were washed, hybridized with Alexafluor 488 and Alexafluor 595 conjugated secondary antibodies (Jackson ImmunoResearch, West Grove, PA) for 90 minutes at room temperature, washed again, and cover slipped in mounting medium with DAPI for nuclear visualization (Vector Labs, Burlingame, CA). Slides were visualized using an Olympus BX51 microscope and CX9000 camera. Controls consisted of irrelevant primary and secondary only conditions.

### Immunohistochemistry

Three or four micron sections of human omentum were subject to immunohistochemistry using the aforementioned primary antibodies, as previously described^23^. To quantify RNase 3 reactive cells in omentum, slides were scanned at 20x magnification (NanoZoomer, Hamamatsu, Japan) and analyzed with Aperio ImageScope software (Leica, Wetzlar, Germany). Large vessels were excluded. The Positive Pixel Count Algorithm was used to determine the fraction of RNase3+ cells among total cell nuclei, expressed as a percentage.

### Statistical analysis

ROC curves, area under the curve, and indicated statistical analyses were performed using GraphPad (La Jolla, CA). In all cases, *p* < 0.05 was considered statistically significant.

## Supplementary information

Supplementary Data
