# Supplementary material for: Analysis of the Ribonuclease A Superfamily of Antimicrobial Peptides in Patients Undergoing Chronic Peritoneal Dialysis
Source: Sci Rep. 2019 May 23;9:7753. doi: 10.1038/s41598-019-44219-x (PMC6533318; doi:10.1038/s41598-019-44219-x)
Supplement: Supplementary file 1 — Supplementary Data [file 41598_2019_44219_MOESM1_ESM.pdf]

Analysis of the Ribonuclease A Superfamily of Antimicrobial Peptides in Patients Undergoing Chronic Peritoneal Dialysis

Neha Dhingra Pottanat[1]; Amy C. Brook[2], Maria Bartosova[3], Hanna Cortado[4], Sudipti Gupta[4], Birong Li[4], Ashley R. Jackson[4], Martin Vonau[5], Shira Cohen[4], Maria Ferrara[6], Christina B. Ching[4,7], John David Spencer[4,8], Annelie Brauner[9], Donald J. Fraser[2,10], Claus Peter Schmitt[3], Matthias Eberl[2], Rose Ayoob[11*], Brian Becknell[4,8]*

[1]Division of Nephrology, Department of Pediatrics, Riley Children's Hospital and Indiana University School of Medicine, Indianapolis, Indiana, USA; [2]Division of Infection and Immunity, School of Medicine and Systems Immunity Research Institute, Cardiff University, Cardiff CF14 4XN, United Kingdom; [3]Division of Pediatric Nephrology, Center for Pediatric and Adolescent Medicine, University of Heidelberg, Heidelberg, Germany; [4]Center for Clinical and Translational Research, The Research Institute at Nationwide Children's Hospital, Columbus, OH, USA; [5]Department of Pediatrics and Internal Medicine, Ohio State University College of Medicine, Columbus, OH, USA; [6]Division of Neonatology, Department of Pediatrics, Nationwide Children's Hospital, Columbus, OH, USA; [7]Division of Pediatric Urology, Department of Surgery, Nationwide Children's Hospital, Columbus, OH, USA; [8]Division of Nephrology, Department of Pediatrics, Nationwide Children's Hospital, Columbus, OH, USA; [9]Department of Microbiology, Tumor and Cell Biology, Division of Clinical Microbiology, Karolinska Institutet and Karolinska University Hospital, Stockholm, Sweden; [10]Wales Kidney Research Unit, Cardiff University, Cardiff, United Kingdom; [11]Division of Nephrology, Department of Pediatrics, Charleston, WV, USA

Running headline: Antimicrobial ribonucleases in peritoneal dialysis

*** Correspondence to:**

Rose Ayoob, M.D.
Women and Children's Hospital
Medical Office Building, 830 Pennsylvania Ave., Suite 103
Charleston, WV 25302 USA
Fax: 304-388-1577
Tel: 304-388-1552
Email: Rose.Ayoob@camc.org

Or Brian Becknell, M.D., Ph.D.
Nationwide Children's Hospital
700 Children's Drive, W308
Columbus, OH 43205 USA
Fax: 614-722-3122
Tel: 614-355-2762
Email: brian.becknell2@nationwidechildrens.org

Table S1. Clinical characteristics of study population.

Sample	Sample type	Age (yr)	Dialysis vintage (yr)	# Total peritonitis episodes	Episode # under evaluation	Bacteriology	ESKD diagnosis	Sex	Age at PD initiation	PD Modality
1	Peritonitis	72.6	3.08	5	5	*Staphylococcus aureus*	Focal segmental glomerulosclerosis	M	69.5	CAPD
	Stable	73.6	4.06		[redacted]					
2	Peritonitis	50.1	2.80	3	1	*Staphylococcus aureus*	Right nephrectomy	M	47.2	CAPD
	Stable	50.7	3.46		[redacted]					
	Stable	51.1	3.82		[redacted]					
	Peritonitis	51.2	3.89		2	Coagulase negative *Staphylococcus*				
	Stable	51.5	4.18		[redacted]					
	Peritonitis	52.2	4.93		3	*Staphylococcus aureus*				
3	Peritonitis	77.0	2.45	1	1	*Escherichia coli*	Diabetic nephropathy	M	74.5	CAPD
	Stable	77.8	3.21		[redacted]					
4	Peritonitis	67.6	1.62	2	1	*Streptococcus sanguinis*	CKD, Cause not specified	F	66.0	CAPD
	Stable	68.2	2.17		[redacted]					
5	Peritonitis	65.0	0.63	7	3	*Staphylococcus aureus*	IgA nephropathy	M	65.3	CAPD
	Stable	65.6	0.25		[redacted]					
	Stable	66.0	0.68		[redacted]					
	Peritonitis	67.6	2.25		7	Culture not sent				
6	Stable	73.8	3.51	2	[redacted]		Diabetic nephropathy	M	70.2	CAPD
	Peritonitis	73.9	3.66		1	*Escherichia coli*				
	Peritonitis	74.0	3.69		2	Culture negative				
	Stable	74.3	3.99		[redacted]					
7	Peritonitis	78.1	2.26	4	2	Coagulase negative *Staphylococcus*	Hypertensive renovascular disease	M	75.7	CAPD
	Peritonitis	79.4	3.59		3	Coagulase negative *Staphylococcus*				
	Stable	80.1	4.31		[redacted]					
	Stable	80.4	4.58		[redacted]					
8	Peritonitis	79.8	0.94	1	1	*Enterobacter* species	CKD, Cause not specified	M	78.8	CAPD
	Stable	80.9	2.11		[redacted]					
9	Peritonitis	64.0	0.02	1	1	Culture negative	CKD, Cause not specified	M	63.9	CAPD
	Stable	65.0	1.02		[redacted]					
10	Peritonitis	75.2	1.07	5	1	Coagulase negative *Staphylococcus*	Ischemic nephropathy	M	74.1	CAPD
	Peritonitis	75.5	1.36		2	Culture negative				
	Stable	76.1	2.00		[redacted]					
	Peritonitis	76.3	2.23		3	*Corynebacterium jeikeium*				
	Stable	76.8	2.65		[redacted]					
	Stable	76.9	2.76		[redacted]					
	Peritonitis	77.0	2.94		4	Coagulase negative Staphylococcus				
	Stable	77.6	3.46		[redacted]					
	Stable	77.8	3.70		[redacted]					
	Peritonitis	77.1	2.99		5	Coagulase negative *Staphylococcus*				
11	Peritonitis	73.0	0.97	6	2	Coagulase negative *Staphylococcus*	Traumatic or surgical renal loss	M	72.0	CAPD
	Peritonitis	73.1	1.06		3	Coagulase negative *Staphylococcus*				
	Peritonitis	73.4	1.39		4	*Staphylococcus aureus*				
	Peritonitis	73.9	1.88		5	Coagulase negative *Staphylococcus*				
	Stable	74.0	1.97		[redacted]					
	Stable	74.1	2.09		[redacted]					
	Stable	74.4	2.40		[redacted]					
	Stable	74.7	2.65		[redacted]					
12	Stable	83.2	0.60	2	[redacted]		Polycytic kidney disease (AD - unspecified)	M	82.5	CAPD
	Stable	85.0	2.38		[redacted]					
	Peritonitis	85.7	3.14		1	*Escherichia coli*				
	Peritonitis	86.1	3.48		2	*Escherichia coli*				CCPD
13	Stable	73.0	1.29	1	[redacted]		CKD, Cause not specified	F	71.6	CAPD
	Peritonitis	73.3	1.63		1	Alpha hemolytic *Streptococcus*				
	Stable	73.9	2.27		[redacted]					
14	Peritonitis	59.0	0.38	2	1	Group B *Streptococcus*	Idiopathic membranous nephropathy	F	58.6	CAPD
	Peritonitis	59.4	0.81		2	*Neisseria* species				
	Stable	60.2	1.56		[redacted]					
	Stable	60.4	1.83		[redacted]					
15	Peritonitis	33.1	0.24	3	1	*Streptococcus sanguinis*	Renal dysplasia	F	32.8	CAPD
	Stable	33.4	0.60		[redacted]					
	Stable	33.6	0.75		[redacted]					
	Peritonitis	35.8	2.96		3	*Staphylococcus aureus*				
16	Peritonitis	58.4	0.80	2	1	*Escherichia coli*	Polycytic kidney disease (AD - unspecified)	F	57.6	CAPD
	Stable	58.7	1.08		[redacted]					
	Stable	59.1	1.50		[redacted]					
	Peritonitis	59.3	1.73		2	*Acinetobacter ursingi*				
17	Peritonitis	74.5	0.44	2	1	Coagulase negative *Staphylococcus*	Ischemic nephropathy	M	74.1	CCPD
	Peritonitis	74.6	0.52		2	Coagulase negative *Staphylococcus*				
	Stable	75.4	1.25		[redacted]					
18	Peritonitis	26.5	0.33	1	1	Coagulase negative *Staphylococcus*	IgA nephropathy	M	26.2	CAPD
	Stable	26.8	0.58		[redacted]					
19	Peritonitis	42.5	0.80	1	1	Alpha hemolytic *Streptococcus*	Idiopathic membranous nephropathy	F	41.7	CAPD
	Stable	43.1	1.38		[redacted]					
20	Peritonitis	51.9	0.07	2	1	Alpha hemolytic *Streptococcus*	Diabetic nephropathy (DM2)	F	51.8	CAPD
	Stable	52.2	0.37		[redacted]					
21	Stable	52.6	1.05	3	[redacted]		CKD, Cause not specified	F	51.5	CAPD
	Peritonitis	52.7	1.18		2	Coagulase negative *Staphylococcus*				
22	Stable	14.4	0.59	0	[redacted]		CKD, Cause not specified	F	13.8	CCPD
23	Stable	15.4	0.42	0	[redacted]		Posterior urethral valves	M	15.0	CCPD
24	Stable	2.9	0.25	0	[redacted]		CKD, Cause not specified	M	2.7	CCPD
25	Stable	10.1	5.66	1	[redacted]		Renal cystic dysplasia	F	4.4	CCPD
26	Stable	21.1	1.25	0	[redacted]		Anti-Glomerular Basement Membrane Disease	M	19.8	CCPD
27	Stable	4.3	4.08	3	[redacted]		Posterior urethral valves	M	0.3	CCPD
	Peritonitis	5.4	5.38		3	Coagulase negative *Staphylococcus*				

Figure S1. Peritoneal fluid RNase concentrations in adult and pediatric chronic PD patients in the absence of infection. In 21 adult and 6 pediatric patients, baseline RNase 3 levels were not significantly different (n.s.). Compared to adult levels, pediatric RNase 6 levels were lower, and RNase 7 levels were higher (** $p < 0.0001$, Mann-Whitney test).

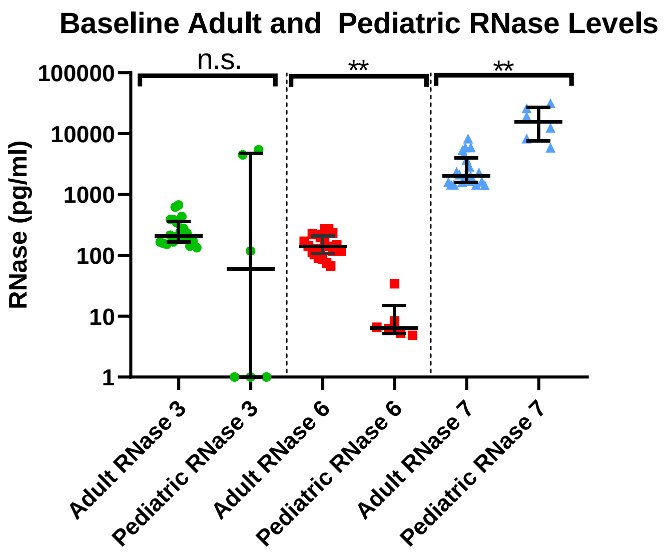

Figure S1

Figure S2. Increased RNase 3 expression in patients with recurrent peritonitis. RNase3 levels increased with each recurrent peritonitis episode (P), compared to intervals obtained at least 3 months after peritonitis (Stable, S).

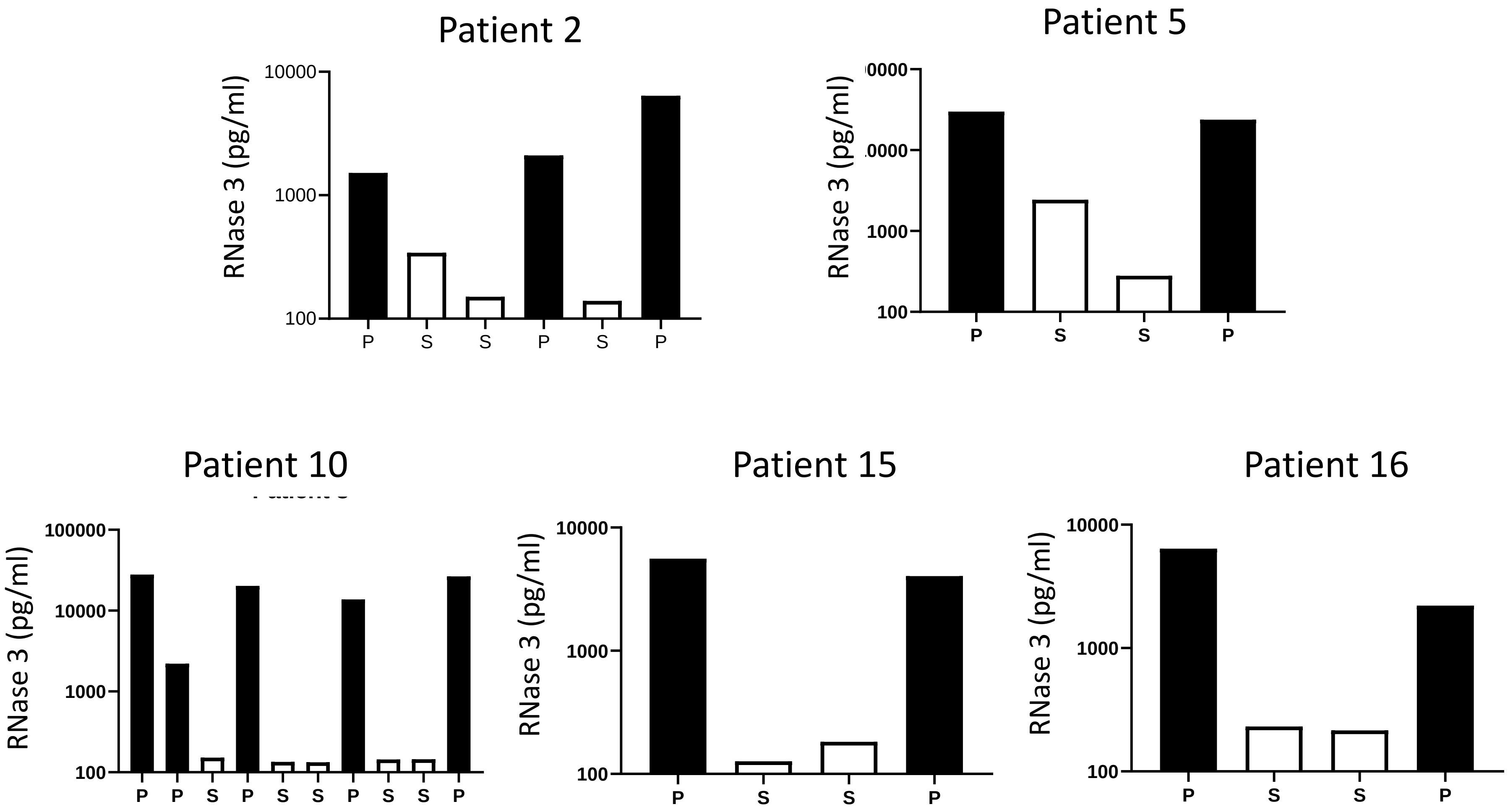

Figure S2
